# myKaryoView: A Light-Weight Client for Visualization of Genomic Data

**DOI:** 10.1371/journal.pone.0026345

**Published:** 2011-10-26

**Authors:** Rafael C. Jimenez, Gustavo A. Salazar, Bernat Gel, Joaquin Dopazo, Nicola Mulder, Manuel Corpas

**Affiliations:** 1 European Bioinformatics Institute, Wellcome Trust Genome Campus, Hinxton, Cambridge, United Kingdom; 2 Computational Biology Group, Department of Clinical Laboratory Sciences, IIDMM, University of Cape Town, Cape Town, South Africa; 3 Universitat Politècnica de Catalunya, Barcelona, Spain; 4 Centro de Investigación Príncipe Felipe, Valencia, Spain; 5 Functional Genomics Node (INB), CIPF, Valencia, Spain; 6 Wellcome Trust Sanger Institute, Wellcome Trust Genome Campus, Hinxton, Cambridge, United Kingdom; Instituto de Ciencia de Materiales de Madrid - Instituto de Biomedicina de Valencia, Spain

## Abstract

The Distributed Annotation System (DAS) is a protocol for easy sharing and integration of biological annotations. In order to visualize feature annotations in a genomic context a client is required. Here we present myKaryoView, a simple light-weight DAS tool for visualization of genomic annotation. myKaryoView has been specifically configured to help analyse data derived from personal genomics, although it can also be used as a generic genome browser visualization. Several well-known data sources are provided to facilitate comparison of known genes and normal variation regions. The navigation experience is enhanced by simultaneous rendering of different levels of detail across chromosomes. A simple interface is provided to allow searches for any SNP, gene or chromosomal region. User-defined DAS data sources may also be added when querying the system. We demonstrate myKaryoView capabilities for adding user-defined sources with a set of genetic profiles of family-related individuals downloaded directly from 23andMe. myKaryoView is a web tool for visualization of genomic data specifically designed for direct-to-consumer genomic data that uses publicly available data distributed throughout the Internet. It does not require data to be held locally and it is capable of rendering any feature as long as it conforms to DAS specifications. Configuration and addition of sources to myKaryoView can be done through the interface. Here we show a proof of principle of myKaryoView's ability to display personal genomics data with 23andMe genome data sources. The tool is available at: http://mykaryoview.com.

## Introduction

Advances in genome sequencing and screening technologies are producing genomic data at an unprecedented scale. Direct-to-consumer (DTC) genetic testing is also becoming relatively successful at attracting people who would like to have their genetic profile genotyped. Genetic profiles, however, provide little biological insight unless they are compared to other relevant data sources. In order to extract any meaning from genetic profiles containing single nucleotide polymorphisms (SNPs), copy number variations (CNVs) or specific genomic variants, raw genome data should be analysed in the context of other genes and annotations. A wealth of databases and annotation resources are available over the Internet with data that can help enrich results obtained in direct-to-consumer tests. Of particular relevance are resources such as HGNC [1] that gives unique and coherent nomenclature for genes, which can then be mapped to specific genome coordinates, the On-line Mendelian Inheritance in Man (OMIM) database [2] that characterises genes and syndromes involved in diseases inherited in a Mendelian fashion, the Database of Genomic Variants [3] that collects mostly CNVs reported in normal individuals, or the Database of Somatic Mutations in Cancer (COSMIC) [4]. These resources may prove useful when determining the potential genetic origin of specific traits in the person. In order to integrate genetic data together into a single interface it is usually necessary to locate the database or resource, download the data, write a specific parser for it and insert matched snippets of information into another database that can be queried against. In addition, this process of retrieval of data has to be periodically repeated to make sure that the retrieved information is up-to-date.

The Distributed Annotation System (DAS) is a widely used protocol for the exchange of biological information using XML [5]. DAS has the advantage of dramatically facilitating the process of integration of disparate biological annotations, providing one single mode of access via a RESTful web service. A DAS registry containing published DAS resources allows the browsing of relevant DAS sources. There are DAS sources for many different organisms and reference assemblies. By selecting a reference assembly, human variation data sources listed in the DAS registry can be easily integrated and mapped via a common coordinate reference system.

Genome visualization resources like Ensembl [6] rely heavily on data sources available via DAS. Ensembl is a very sophisticated system used for a great variety of species with many different kinds of data, allowing relatively complex integration and search queries. Ensembl is particularly useful for integration of many data sources, shown as tracks at different regions in the genome. Despite Ensembl's and other browsers' [7], [8] ability to show different levels of annotation, every time coordinates are changed, the page needs to be refreshed to reload all the information with no simultaneous visualisation of different zoom levels. Furthermore, visualisation of karyotypes is either disallowed or too impractical to allow sufficient interactivity for the user when wanting to view information at the genome level. Therefore, we designed a flexible tool for visualisation of genomic DAS annotations that is complementary to existing genome browsers and yet specifically tailored to visualization of DTC genomic data. This tool, named *myKaryoView*, allows easy navigation between different levels of scale in three integrated ‘views’ that interact with each other. myKaryoView is thus a client that operates under a single interface and constitutes a “one stop shop” for simple and rapid queries, not requiring much knowledge of either bioinformatics tools or information technology in order to operate it. myKaryoView provides three views: karyotype, chromosome and zoom. Once the data is rendered, further requests are not needed to navigate freely within the region (unless zooming out). Each DAS data source is represented in different tracks with annotations that are clickable and provide further information about that particular feature and links to the relevant sources. Links back to Ensembl from any selected region allow automatic loading of the region in the Ensembl browser. A simple interface is provided to query myKaryoView by SNP id, gene name or genomic region as well as selection of user-defined DAS sources.

myKaryoView has been specifically configured to provide an integrated view of sources for analysis of DTC genomic data. But it can also be used for visualization of any genomic DAS data feature as long as it is in the same coordinate of reference. myKaryoView is able to provide a genome-wide perspective and easy navigation between different levels of detail with no need for refreshing the page, giving an overall enhanced visualization experience for genome analysis. myKaryoView's search interface and navigation widgets provide a variety of options that may help shed light on interpretation of genetic profiles and personal genomics data in general. myKaryoView does not require a lot of specialised knowledge in order to operate its simplest functions, although biological knowledge is required in order to appropriately understand the data sources integrated by default in the browser. Its human variation genome-focused configuration of views and sources of data make it appropriate for direct-to-consumer genome data analysis.

## Results

### Genome Navigation

Several widgets are provided for easy navigation and further exploration of retrieved annotation features. The chromosome view has a slider widget for zooming in or out of selected chromosomal regions. Moving the right and left slider to the desired region to be zoomed in and clicking “Zoom” refreshes the zoom view to this region. Any band in the chromosome view can also be clicked on and automatically displayed in the zoom view. Annotations are shown in different tracks with their respective legends at the top of the view. The legend shows the colour in which a particular annotation is displayed and their number in parentheses. Clicking on the legend, a popup window appears with a link “Display Annotations in Ensembl”. This popup window also provides a link for viewing of the original data as it is retrieved via DAS. Any of the tracks can be selected/deselected by checking/unchecking the check box next to the legend. Any chromosome in the karyotype view, if clicked on, is automatically reloaded in the chromosome and zoom views. By clicking on any of the features, another popup window appears with more specific annotation about the feature and, where applicable, links to the original source. All of the data for any of the displayed sources can also be downloaded if the legend is clicked and the ‘Show Original Source’ link is selected. A new tab is thus opened with a formatted version of the raw data as retrieved from the server. This is especially useful if further analyses are sought with the visualised data, as this view allows easy copying and pasting of data as text, containing all annotations for a particular data source. Once the search query is performed there is little need to go back to the initial interface for navigation across any region in the genome.

We provide proof-of-principle results to show that a) it is possible to add user-defined data for visualisation of direct-to-consumer genome data and b) it can be used as a stand-alone genome browser for visualization of human genetic variation data.

#### a) Personal Genomics Family Case

**Figure 1:**
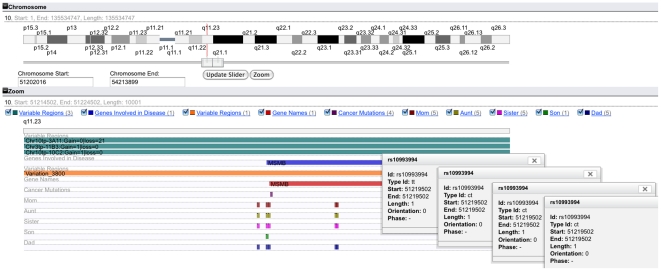
Searching myKaryoView by SNP. The rs10993994 SNP was identified in a 23andMe report to contribute most to the disease risk of prostate cancer in a customer, implicated by the MSMB gene. In addition to the customer, 4 additional members of his family uploaded their genotypes into myKaryoView for elucidation of how this particular SNP genotype was inherited. Popup windows for the SNP are shown in this order: son, mother, father and sister. The Type Id field shows their genotype for this position in their genome, TT, CT, CT, CT respectively. The aunt (not shown) has a CC genotype for this position.

A 23andMe customer (“son”) wanted to find more information about the genetic causes leading to his increased risk of prostate cancer. An analysis of his genome profile yields a 28.1% risk of developing the cancer as opposed to the 17.8% average risk in males. This risk is calculated analysing the genotypes of 12 SNPs. The SNP marker rs10993994 shows the greatest risk among the 12 reported markers, a 1.3 times increased odds. This SNP is located in 10q11, near the *MSMB* gene and the effects of the identified allele (T) have been shown to decrease its expression levels [9], thus decreasing its cancer suppressor function. Having no history of prostate cancer in close relatives, 4 family members for the same customer were genotyped (mother, father, sister and aunt) and their data was made into DAS source in order to compare the pattern of inheritance between different family members for this SNP using myKaryoView. All these individual's genome profiles, were downloaded from 23andMe and a DAS source was created using easyDAS [10]. The resulting data sources were held privately in his newly created easyDAS account. The DAS URLs for the private sources were pasted into the myKaryoView interface and all other sources were selected to display in the zoom view. The ‘rs10993994’ SNP was typed in the query search box. Once results were returned, the zoom view was centred around the rs10993994 SNP±100 Kb of the SNP position (51219502) (Figure 1). Next, the pattern of inheritance for this SNP was elucidated by clicking on the SNP in the other members of the family analyzed. Clicking on the rs10993994 SNP feature in each family member track triggers a popup window with information about the SNP, among them the Type Id, which provides the actual genotype for that position. It was found that the father and the mother both have CT, with no risk associated with this variant, while the son inherited a T from each parent, producing the TT genotype with increased disease risk. The density of the number of analyzed SNPs is also clearly depicted by myKaryoView, showing that the son has fewer analyzed SNPs for this region than the other members of the family. This is due to the fact that a new SNP chip version was used with double the number of features for the other members of the family. Furthermore, it was noted that the start position of the MSMB gene is 51219559, only 57 bp after the SNP position. The Cancer Mutations track contains four reported mutations for MSMB (MSMB:ENST00000358559), indicative of the involvement of this gene in cancer, but all of them within the gene's exons. The overlap of four normal CNVs in the two Variable Region tracks show this is a hugely variable region. Through such close proximity of this SNP to the MSMB gene and the recorded variation, findings are consistent with the observation that an allele variant found in the 23andMe analysis could potentially have an effect on the promoter region of MSMB, suggesting a causal variant at 10q11 that confers increased risk of prostate cancer. myKaryoView is thus able to provide a convenient visualization interface for easily navigating and querying any SNP the DTC customer might be interested in. Although it is not a substitute for the interpretation of the genomic data tested in an individual, it provides a contextualization and perhaps a solution to start exploring data sources that might help elucidate phenotypic associations. By integrating the genotypes of different family members, myKaryoView provides a convenient search interface for visualization of analyzed feature density and ascertaining patterns of inheritance in related individuals.

#### b) Human Genetic Variation Analysis Case

myKaryoView can also serve the purposes of a generic genome browser in addition to its application to personal genomics shown above. We demonstrate its generic capabilities in this second use case.

Deletion of the 15q11 region has been related to Prader-Willi and Angelman Syndromes [11], [12], [13]. This region roughly corresponds to the interval 20000000–24000000 in chromosome 15. According to the DECIPHER database [14], deletion of this region results in mental retardation and developmental delay. In order to explore genes likely to affect the patient phenotype, myKaryoView was utilised to look for genes involved in disease that overlapped this region and were reported to be involved in Mendelian disease inheritance, as well as regions of normal copy number variation. All available DAS sources were selected to display in the zoom view as tracks. In the input search box, the string ‘15∶20000000,24000000’ was entered.

**Figure 2:**
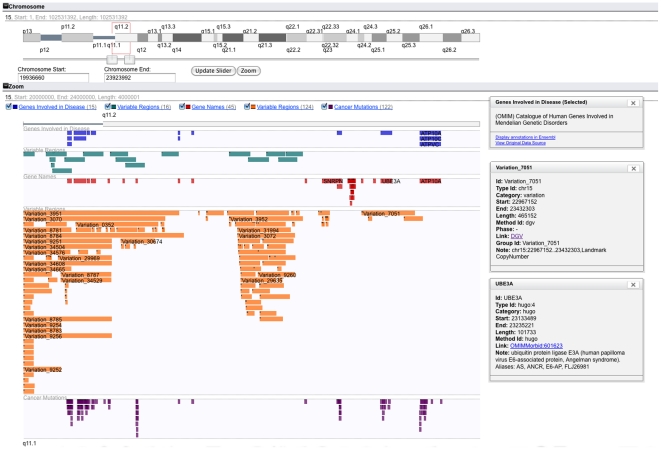
Chromosome Location Search. Searching 15∶20000000,24000000 displays part of the 15q11 chromosomal band in zoom view. All listed DAS sources (gene names, genes involved in disease, cancer mutations and variable regions) were selected, choosing zoom and track visualisation options. The total number of features per track is shown next to the legend in parentheses. Forty five genes lie in selected intervals. Clicking on the ‘Genes Involved in Disease’ legend, a popup window appears providing links to the region in Ensembl and the original raw data that can be easily cut and pasted. The UBE3A gene bar is clicked and another popup window appears with links to further information. Variation 7051 reported by DGV is also clicked.

Figure 2 shows results obtained from this search query. Fifteen genes involved in disease were retrieved from a total of forty-five genes in this region. Clicking on the ‘Genes Involved in Disease’ legend, a popup window appears with the option ‘View Original Data Source’, which triggers a new tab when clicked, with the original data source as obtained from the source (OMIM). myKaryoView results show greater density of ‘normal’ CNVs in gene desert regions. One notable exception to this pattern is Ubiquitin protein ligase E3A, a gene reported to produce an abnormal phenotype in ubiquitin-mediated protein degradation during brain development when this region is deleted [15]. This gene overlaps with Variation 7051 in DGV, a 0.4 Mb deletion reported by de Smith et al (2007) [16], using an Agilent 185 k CGH Array. We notice that a nearby cluster of genes of the SNORD family are included in Variation 7051 but are outside the myKaryoView graph, showing that genome coordinates for different sources may vary slightly. Moreover, due to array resolutions, it is possible that genes reported to be included in a normal CNV may, in reality, be misreported as such due to variable resolution of CNV detection methods.

## Discussion

### User Interaction Capabilities

A selection of human variation data sources is available by default in myKaryoview's website. These are DAS sources known to be relevant for genome variation analyses: HGNC Genes, OMIM, COSMIC, DGV and Redon clones [17]. In order to make them intelligible to lay users we have named them as: gene names, genes involved in disease, cancer mutations and variable regions. To make a request, at least one source has to be selected or uploaded. Input parameters are provided for users in the query interface for the following options: a) type of feature visualisation device (mark, track, line or chart), b) level of detail (views) in which to visualise the selected data source (karyotype, chromosome or zoom) and c) an input text box for specification of user-defined DAS sources. A URL with a valid DAS source address is required for user-defined DAS sources. Currently the interface is designed to allow one user-defined source, but this capability will be expanded in the near future to allow simultaneous visualization of several family member genomes. Existing genomic DAS sources available in the DAS registry are suitable for the input text box, as long as the source is built on the same reference assembly as the one used by myKaryoView (currently *Homo sapiens* NCBI36). Alternatively, user-defined DAS sources can be created. easyDAS is a tool that simplifies the process of DAS source creation and is able to convert genomic data from a variety of formats into a valid source which can be public or private. The created URL for the DAS source can then be added to the myKaryoView interface and visualised as any other data source.

The final requirement to run a query concerns entering the actual region or gene to be visualized. myKaryoView allows the search of any chromosomal location in the genome. A chromosome, and a start and end position separated by a colon and comma respectively (e.g. 1∶2000000,3000000), constitutes the valid format for a chromosome location search. Searching can also be accomplished using valid HGNC gene names (also known as symbols). If searching by genes, the user should start by typing any character and selecting the appropriate gene name from a drop-down box that appears with suggestions. Once a gene is chosen from the list, one can hit ‘Submit Query’. Results will appear as they are retrieved, with the zoom view centred on the start and end coordinates of the entered chromosomal location or the start and end position of the chosen gene.

### Availability and Future Directions

The software can be freely used through the website http://mykaryoview.com. The source code is also available and can be downloaded as a web application under an Apache 2.0 license. We anticipate that direct-to-consumer genetic test customers would benefit from using myKaryoView by further exploring and discovering new insights not available in their test results. We envisage myKaryoView as a simple-to-use complementary resource to more sophisticated genome browsers.

## Methods

### Implementation and Design

myKaryoView is a light-weight client specifically designed to browse genomic data available via DAS with no data stored locally. Currently myKaryoView is configured to display *Homo sapiens* NCBI36 genome assembly data features, but it can also be used with alternative assemblies or even different organisms by simply modifying the configuration file in its source code, for which a manual is provided for advanced users. Although the NCBI36 assembly is not the most up-to-date human assembly, we chose this one to maintain compatibility with the raw bed file data provided by 23andMe, which currently is NCBI36. The source code is freely available for download under a CCA-SA license and authors welcome requests of support from potential collaborators. The fact that it is mainly written in cross-browser javascript code means that myKaryoView should work in every major browser and if installed externally it should work automatically in a web server directory. Currently we have tested myKaryoView in Internet Explorer 6 and, 7, Mozilla Firefox and Chrome. myKaryoView works well when the total number of features to be rendered in one go is in the order of several thousand. Although whole genome requests can, in principle, be handled by myKaryoView, if too many features are requested, this may overwhelm the computer's RAM capacity. Another limitation lies in the time it takes for data retrieval. Since all the data has to be retrieved from the Internet, slow Internet connections may negatively impact the user's experience. For most queries however, retrieval and rendering of data should be achieved in a few seconds.
